# The Relationship Between Job Stress and Job Satisfaction of Primary and Secondary School Physical Education Teachers: A Moderated Mediation Model

**DOI:** 10.3390/bs16050737

**Published:** 2026-05-09

**Authors:** Xiaolong Zhang, Ziyi Zhang, Shan Huang, Weijie Gao, Kexiang Yang, Yufeng Han, Hyun-Chul Jeong

**Affiliations:** Department of Physical Education, Jeonbuk National University, Jeonju-si 54896, Republic of Korea; 202555102@jbnu.ac.kr (X.Z.); zzy1060365890@163.com (Z.Z.); huangshanjbnu@163.com (S.H.); gaowj@jbnu.ac.kr (W.G.); 202455495@jbnu.ac.kr (K.Y.); 202555103@jbnu.ac.kr (Y.H.)

**Keywords:** physical education teachers, job stress, job satisfaction, job burnout, psychological resilience

## Abstract

This study examined the relationship between job stress and job satisfaction among primary and secondary school physical education teachers, with job burnout as a mediator and psychological resilience as a moderator. A total of 444 Chinese physical education teachers completed the Questionnaire on Occupational Stress and Stressors for Primary and Secondary School Physical Education Teachers, the Teacher Job Satisfaction Questionnaire, the Maslach Burnout Inventory-Educator Survey (MBI-ES), and the Teacher Psychological Resilience Scale. The results showed that: (1) job stress negatively predicted job satisfaction (*β* = −0.397, *p* < 0.001); (2) job burnout partially mediated the relationship between job stress and job satisfaction (indirect effect = −0.203); (3) psychological resilience moderated the relationship between job stress and job burnout (*β* = −0.12, *p* < 0.001). These findings support the mediating role of job burnout and show that psychological resilience weakens the positive association between job stress and job burnout, such that job stress affects job satisfaction both directly and indirectly through burnout, with a stronger indirect effect among teachers with lower psychological resilience.

## 1. Introduction

Primary and secondary school physical education teachers (hereinafter referred to as “physical education teachers”) play a vital role not only in students’ physical development and health, but also in their psychological well-being and social adjustment (Trigueros et al., 2019; Kliziene et al., 2021). However, physical education teachers often work within a school environment in which physical education is undervalued, their professional status is marginalized, and instructional time is vulnerable to being reduced or occupied by academic subjects (Richards et al., 2017a; Kougioumtzis et al., 2011; Ferry & Westerlund, 2023; Yan & Dai, 2025). In practice, physical education teachers not only carry heavy teaching loads, but also devote considerable effort to classroom management and handling students’ disruptive behaviors (Sáenz-López et al., 2011). They must also ensure student safety and health (Eirín-Nemiña et al., 2022), while insufficient sports teaching equipment further increases the difficulty of practical instruction (Sáenz-López et al., 2011; Ma’mun et al., 2025). Compared with teachers of many other subjects, physical education teachers may therefore face not merely a greater amount of stress, but a more distinctive configuration of job demands and resource constraints, in which instructional, physical, organizational, and status-related pressures are intertwined (Richards et al., 2017a; Sáenz-López et al., 2011; Ferry & Westerlund, 2023; Yan & Dai, 2025). This suggests that, in the case of physical education teachers, job stress may be more readily associated with resource depletion and subsequent job burnout, thereby affecting job satisfaction. In the long term, the cumulative effects of heavy workload and multiple stressors may lead to physical and psychological fatigue, and, when prolonged, may further undermine teachers’ mental and physical well-being (Michie, 2002; Yan et al., 2025), thereby leading to the deterioration of job satisfaction (Richards et al., 2017b), and may even contribute to increased teacher attrition (Zhou et al., 2025). Such prolonged occupational strain may gradually weaken physical education teachers’ professional identity and work engagement, ultimately affecting student development. Therefore, examining the relationships among job stress, job burnout, and job satisfaction among primary and secondary school physical education teachers is theoretically meaningful not merely as a repeated validation in another teacher subgroup, but as a way to clarify whether these relationships operate within a more resource-constrained and structurally marginalized teaching context. Such an examination may also provide empirical evidence for optimizing their working environment and informing stress intervention programs, while offering practical value for improving career retention, occupational well-being, teaching quality, and adolescents’ physical and psychological development.

## 2. Literature Review and Hypothesis Development

Although the foregoing discussion highlights the multiple occupational pressures faced by physical education teachers, these challenges alone do not fully explain how job stress translates into lower job satisfaction. To better understand this process, a theoretical perspective is needed to clarify the psychological mechanism through which job stress influences occupational outcomes. In this regard, resource-based perspectives provide a useful lens for understanding how work demands may deplete teachers’ psychological resources and ultimately affect their job-related well-being. Against this background, although previous studies have documented the stressful working conditions of physical education teachers, less attention has been paid to whether this process operates through resource depletion and whether personal resources may weaken such effects. In the present study, the JD-R model is treated as the overarching theoretical framework for explaining how job demands are associated with burnout and job satisfaction, while COR theory is used as a complementary resource-based perspective to clarify why sustained demands may lead to resource depletion and why personal resources may mitigate such effects. Accordingly, the following hypotheses are proposed within a JD-R and COR framework.

### 2.1. Job Stress and Job Satisfaction

Job satisfaction has long been regarded as an important indicator of teachers’ occupational well-being and professional functioning. In Job Satisfaction, Hoppock (1935) defined job satisfaction as workers’ physiological and psychological attitudes or emotional reactions toward elements of their work environment. Within the educational context, teacher job satisfaction generally refers to teachers’ overall affective and cognitive evaluation of their occupation, the work itself, and the broader working environment in schools (Wanous & Lawler, 1972). From the perspective of Self-Determination Theory, teachers’ job satisfaction is closely related to the extent to which their basic psychological needs for autonomy, competence, and relatedness are supported in the work environment (Deci & Ryan, 1985). Under conditions of sustained job stress, these basic psychological needs may be frustrated, thereby reducing teachers’ job satisfaction. When teachers experience higher levels of job satisfaction, they are more likely to demonstrate greater cognitive, motivational, and emotional investment (Wartenberg et al., 2023). By contrast, job stress emerges when work-related demands exceed an individual’s perceived capacity to cope, thereby triggering a series of negative physiological, psychological, and behavioral responses (Ghasemi et al., 2024). Teacher job stress refers to unpleasant emotional experiences, such as anxiety and depression, arising from personal and environmental factors in educational work (Kyriacou & Sutcliffe, 1977; Agyapong et al., 2022). In daily life, a complete absence of stress is neither realistic nor conceivable. Moderate levels of job stress can stimulate motivation and improve job performance, whereas excessive job stress can cause psychological and physical distress (Selye, 1976). For primary and secondary school physical education teachers, such stress may be particularly salient because their work often involves not only routine instructional tasks, but also physically demanding teaching activities, class management, student safety responsibilities, and additional school-related duties. Under these conditions, persistent job stress is likely to weaken teachers’ positive evaluations of their work and reduce their job satisfaction. From the perspective of the JD-R model, when work demands remain high and are not matched by sufficient job resources, the strain experienced by teachers may undermine their occupational well-being and job-related attitudes. Existing studies have shown that job stress is one of the most important and unavoidable factors affecting teachers’ job satisfaction, exerting a marked negative influence on it (Riyadi, 2015). Given the distinctive configuration of job demands and resource constraints faced by physical education teachers compared with teachers of other subjects, this negative association may be particularly pronounced in this group. Based on the above reasoning, Hypothesis H1 is proposed: job stress negatively predicts job satisfaction among physical education teachers.

### 2.2. Mediating Effect of Job Burnout

Although the above discussion helps explain why job stress may directly reduce job satisfaction, this direct relationship alone does not fully capture the psychological process through which occupational stress affects teachers’ work-related outcomes. One important mechanism may be job burnout. The teaching profession is recognized as one of the most stressful professions (Travers, 2017), and this is a global phenomenon (Skaalvik & Skaalvik, 2017). Under long-term emotional stress and workplace stress, teachers are highly prone to job burnout (S. Li & Wang, 2024). Job burnout refers to a state of emotional, attitudinal, and behavioral exhaustion resulting from prolonged exposure to work stress, which is primarily manifested across three core dimensions: emotional exhaustion, depersonalization, and reduced personal accomplishment (Yang et al., 2014). Job burnout can lead to emotional exhaustion, diminished work enthusiasm, and reduced motivation, thereby impairing teachers’ effectiveness and performance. This process may be especially relevant to physical education teachers, whose work often combines heavy instructional demands, physical workload, student safety obligations, and limited material support. In such a context, prolonged job stress may continuously consume teachers’ psychological resources, making it more difficult for them to maintain enthusiasm, emotional stability, and professional engagement. From this perspective, job burnout can be understood as an important psychological pathway linking job stress to reduced job satisfaction. This mechanism can be further explained by the Conservation of Resources (COR) theory, which suggests that prolonged and repetitive work demands may deplete individuals’ emotional resources, thereby increasing psychological strain and vulnerability to burnout. According to the loss spiral proposition in COR theory, when individuals face stressful situations, they expend resources to cope with them, but if coping fails or the resources required are excessive, stress may escalate further (Hobfoll, 2002). Although conceptually related to COR theory, the Job Demands-Resources (JD-R) model was independently proposed and emphasizes that excessive job demands, when not matched by sufficient job resources, may result in energy depletion and eventually lead to job burnout (Demerouti et al., 2001; Bakker et al., 2003). Taken together, these resource-based perspectives provide a useful explanation for why sustained job stress may gradually erode teachers’ internal resources and contribute to burnout. For physical education teachers, an imbalance between work demands, such as excessive teaching loads, safety responsibilities, and insufficient equipment, and available resources may accelerate psychological resource depletion, making it more difficult to maintain a positive work state and increasing the risk of burnout. With regard to the relationship between job burnout and job satisfaction, existing studies have confirmed that physical education teachers’ job burnout negatively predicts job satisfaction (Panagopoulos et al., 2014). Moreover, a meta-analysis revealed a negative correlation between physical education teachers’ work-related burnout and job satisfaction (Böke & Norman, 2022). Based on this, this study proposes Hypothesis H2: Job burnout mediates the relationship between job stress and job satisfaction among primary and secondary school physical education teachers. Specifically, job stress not only has a direct negative impact on job satisfaction but also indirectly reduces job satisfaction through job burnout.

### 2.3. Moderating Effect of Psychological Resilience

The original JD-R model centered on the influence of job-related characteristics on employees’ occupational mental health. However, subsequent JD-R research has increasingly emphasized the role of personal resources in shaping how employees respond to job demands (Xanthopoulou et al., 2007). In the present study, psychological resilience is examined as a particularly relevant personal resource because it directly reflects individuals’ capacity to adapt to stress and recover from adversity, and may therefore help explain why teachers exposed to similar job demands differ in burnout levels, especially relative to more contextual resources such as school support (Wang et al., 2025). From the perspective of COR theory, psychological resilience may also be understood as a resource that helps individuals resist resource loss, recover more effectively from demanding experiences, and reduce the likelihood that sustained stress develops into burnout. Psychological resilience refers to individuals’ capacity to cope with, adapt to, and recover from stressful, adverse, or traumatic circumstances, and in some cases to develop beyond their original level of functioning (Den Hartigh & Hill, 2022). Individuals with high psychological resilience often demonstrate stronger adaptive abilities and more effective coping capabilities for future uncertainties (Nelson et al., 2007). Existing studies have shown that psychological resilience is an important component of individual resources, and it is an important predictor of teacher job burnout (Richards et al., 2016; S. Li, 2023). Specifically, although higher job stress may generally be associated with higher job burnout among physical education teachers (X. Li et al., 2025), this association is expected to vary according to teachers’ levels of psychological resilience. Teachers with higher psychological resilience may be less vulnerable to the detrimental effects of job stress and therefore may show a weaker positive association between job stress and job burnout, whereas teachers with lower psychological resilience may show a stronger positive association. In this sense, psychological resilience is expected not merely to relate to lower burnout in general, but to moderate the strength of the relationship between job stress and job burnout. According to the resource investment principle of COR theory, psychological resilience, as a preventive personal psychological resource, plays a central role in reducing the depletion of an individual’s resources by enhancing their stress coping capacity before stressors trigger resource loss. Accordingly, drawing on the personal resource perspective within the JD-R model and the resource investment principle of COR theory, Hypothesis H3 is proposed: psychological resilience negatively moderates the positive relationship between job stress and job burnout among primary and secondary school physical education teachers. Specifically, the higher the level of psychological resilience, the weaker the positive predictive effect of job stress on job burnout.

In summary, this study constructed a first stage moderated mediation model to examine whether job burnout mediates the relationship between job stress and job satisfaction, and whether psychological resilience moderates the first stage of this indirect pathway, namely, the association between job stress and job burnout, thereby making the indirect effect conditional on teachers’ levels of psychological resilience.

## 3. Materials and Methods

### 3.1. Participants and Procedures

This study used convenience sampling to recruit primary and secondary school physical education teachers from Henan, Guangdong, Jiangsu, Beijing, and other regions of China, and measured their job stress, job satisfaction, job burnout, and psychological resilience. The questionnaire was distributed via the Wenjuanxing platform through WeChat to heads of primary and secondary school physical education teaching and research groups, teacher WeChat groups, and physical education teacher contacts. Participants were eligible if they were currently engaged in physical education teaching in primary or secondary schools in China and completed the questionnaire voluntarily. No additional exclusion criterion was imposed based on teaching experience or substitute teacher status. Respondents who did not identify themselves as physical education teachers or provided incomplete questionnaires were excluded from the final analysis. Because the questionnaire was distributed through channels targeting primary and secondary school physical education teachers, respondents were identified as physical education teachers based on self-reported occupational information collected in the questionnaire, including teaching stage and other teaching-related background variables.

Before completing the questionnaire, all participants were required to read an online informed consent form, which clearly stated that they could voluntarily withdraw from the study at any time if they experienced any discomfort during the process. All materials and procedures of this study were approved by the Institutional Ethics Committee of Jeonbuk National University (IRB number: KIRD-2025-12-31-J-E-008000) and strictly adhered to the ethical principles outlined in the Declaration of Helsinki. Throughout the research process, participants’ anonymity and data confidentiality were guaranteed, and all participants provided informed consent after fully understanding the study content. A total of 478 questionnaires were distributed, and 444 valid questionnaires were recovered (92.89%). The sample included 160 males (36.00%) and 284 females (64.00%); 65 teachers aged 18–25 (14.64%), 135 aged 26–30 (30.41%), 80 aged 31–40 (18.02%), 105 aged 41–50 (23.65%), and 59 aged 51–60 (13.29%); 288 married (64.86%) and 156 unmarried (35.14%); 201 primary school teachers (45.27%), 182 junior high school teachers (40.99%), and 61 senior high school teachers (13.74%); 399 public school teachers (89.86%) and 45 private school teachers (10.14%); 188 with teaching experience under 5 years (42.34%), 58 with 6–10 years (13.06%), 60 with 11–20 years (13.51%), 94 with 21–30 years (21.17%), and 44 with over 31 years (9.91%); 61 college graduates (13.74%), 309 undergraduates (69.59%), and 74 postgraduates and above (16.67%); 32 third-level teachers (7.21%), 145 second-level teachers (32.66%), 128 first-level teachers (28.83%), 60 associate senior teachers (13.51%), 1 senior teacher (0.23%), and 78 others (17.57%).

### 3.2. Measures

#### 3.2.1. Job Stress

Job stress was measured using the Questionnaire on Occupational Stress and Stressors for Primary and Secondary School Physical Education Teachers, developed by Lin Xiaoqun et al. (Lin et al., 2005). The questionnaire includes 27 items across five dimensions: students, parents, examinations, work, and professional needs. The scale uses a five-point Likert scale (1 = no stress, 5 = extreme stress), where higher scores indicate greater job stress. In this study, Cronbach’s *α* coefficient of the scale was 0.97.

#### 3.2.2. Job Satisfaction

The Teacher Job Satisfaction Questionnaire, developed by Xu Zhiyong and Zhao Zhihong (Xu & Zhao, 2012), was used. It consists of 10 items across two dimensions: intrinsic job satisfaction and extrinsic job satisfaction. The scale uses a five-point Likert scale (1 = strongly disagree, 5 = strongly agree), where higher scores indicate higher job satisfaction. Cronbach’s *α* coefficient of the scale was 0.96.

#### 3.2.3. Job Burnout

The Maslach Burnout Inventory-Educator Survey (MBI-ES) was developed by Maslach et al. (Maslach et al., 2001) and includes 22 items across three dimensions: emotional exhaustion, depersonalization, and reduced personal accomplishment. The scale uses a five-point Likert scale (1 = never, 5 = always). Emotional exhaustion and depersonalization are positively scored, while the reduced personal accomplishment dimension is negatively scored. Higher total scores indicate more severe job burnout. Cronbach’s *α* coefficient of the scale was 0.90.

#### 3.2.4. Psychological Resilience

The Teacher Psychological Resilience Scale, developed by Q. Li et al. (2014), was used. It consists of 13 items across three dimensions: love and dedication to teaching and learning, teacher self-efficacy, and job satisfaction and optimism. The scale uses a five-point Likert scale (1 = strongly disagree, 5 = strongly agree), with higher total scores indicating higher psychological resilience. In this study, Cronbach’s *α* coefficient of the scale was 0.97.

### 3.3. Statistical Processing

The common method bias test was conducted using SPSS 27.0, followed by descriptive statistics and correlation analysis for each variable. The mediating and moderating effects were analyzed using the PROCESS 4.2 macro for SPSS developed by Hayes (Hayes, 2012). The 95% confidence intervals of the mediating and moderating effects were estimated using the bias-corrected percentile Bootstrap method with 5000 resamples. In addition, supplementary analyses were conducted to compare primary and secondary school physical education teachers and to examine whether the proposed model showed a similar pattern across the two groups.

## 4. Results

### 4.1. Common Method Bias Analysis

Harman’s single-factor exploratory factor analysis was used to test for common method bias. The results showed that eight factors had eigenvalues greater than 1, with the maximum variance explained by a single factor being 34.57%, lower than the general empirical criterion of 40%. These results suggest that common method bias was unlikely to be a serious concern in this study (Podsakoff et al., 2003).

### 4.2. Descriptive Statistics and Correlation Analysis

Table 1 presents the means, standard deviations, and Pearson correlations of all variables. The correlation analysis indicated that job stress was positively correlated with job burnout and negatively correlated with job satisfaction and psychological resilience (*r* = 0.53, *p* < 0.01; *r* = −0.40, *p* < 0.01; *r* = −0.26, *p* < 0.01). Job satisfaction was positively correlated with psychological resilience and negatively correlated with job burnout (*r* = 0.75, *p* < 0.01; *r* = −0.50, *p* < 0.01), whereas job burnout was negatively correlated with psychological resilience (*r* = −0.52, *p* < 0.01). Additionally, age group was positively correlated with job satisfaction and psychological resilience and negatively correlated with job burnout (*r* = 0.19, *p* < 0.01; *r* = 0.15, *p* < 0.01; *r* = −0.11, *p* < 0.05). Marital status was positively correlated with job burnout and negatively correlated with job satisfaction and psychological resilience (*r* = 0.10, *p* < 0.05; *r* = −0.17, *p* < 0.01; *r* = −0.13, *p* < 0.01). Teaching experience was positively correlated with job satisfaction and psychological resilience and negatively correlated with job burnout (*r* = 0.16, *p* < 0.01; *r* = 0.13, *p* < 0.01; *r* = −0.11, *p* < 0.05), whereas education level was positively correlated with job burnout (*r* = 0.11, *p* < 0.05). To minimize the confounding effects of demographic variables on the core relationships, age group, marital status, and teaching experience were included as control variables in subsequent analyses, based on the correlation pattern reported in Table 1.

### 4.3. Multicollinearity Test

Because several variables were correlated, multicollinearity was assessed to determine whether it might affect the stability of the results. Job satisfaction was treated as the outcome variable, and job stress, job burnout, and psychological resilience were treated as predictors in this diagnostic analysis. The results showed that the tolerances of all predictor variables (0.55–0.71) were all greater than 0.1, and the Variance Inflation Factor (VIF) ranged from 1.41 to 1.83 (all < 5). These results suggest that multicollinearity was unlikely to be a serious concern in this study.

### 4.4. Test of the Mediating Role of Job Burnout

**Figure 1:**
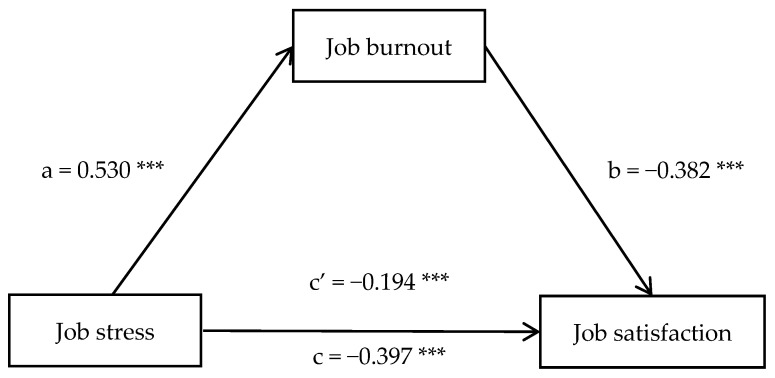
Path diagram of the mediating role among job stress, job burnout, and job satisfaction. *** *p* < 0.001.

After standardizing the variables, Model 4 of the PROCESS macro for SPSS was used to test the mediating effect of job burnout between job stress and job satisfaction while controlling for age group, marital status, and teaching experience. The results showed (Table 2) that job stress negatively predicted job satisfaction (*β* = −0.397, *t* = −9.233, *p* < 0.001). When the mediating variable job burnout was added, job stress positively predicted job burnout (*β* = 0.530, *t* = 13.158, *p* < 0.001), job burnout negatively predicted job satisfaction (*β* = −0.382, *t* = −8.029, *p* < 0.001), and job stress still negatively predicted job satisfaction (*β* = −0.194, *t* = −4.095, *p* < 0.001). Thus, job burnout played a partial mediating role between job stress and job satisfaction. The bias-corrected Bootstrap method (with 5000 resamples) was used to estimate the 95% confidence interval of the mediating effect as [−0.264, −0.144]. The indirect effect (−0.203) accounted for 51.13% of the total effect (−0.397), indicating that job burnout plays a substantial mediating role in the association between job stress and job satisfaction in this group. The path diagram is shown in Figure 1.

### 4.5. Test of the First-Stage Moderated Mediation Effect

According to the suggestions of Wen Zhonglin and Ye Baojuan (Wen & Ye, 2014), after standardizing all continuous variables, Model 7 of Hayes’ PROCESS macro was used to test whether psychological resilience moderated the first stage of the indirect effect of job stress on job satisfaction through job burnout, while controlling for age group, marital status, and teaching experience. The results showed (Table 3) that after controlling for age group, marital status, and teaching experience, Equation (1) was significant overall (*F*_(6,437)_ = 61.97, *p* < 0.001). Job stress (*β* = 0.49, *p* < 0.001) and psychological resilience (*β* = −0.42, *p* < 0.001) both predicted job burnout, and the interaction term of job stress and psychological resilience also predicted job burnout (*β* = −0.12, *p* < 0.001). Equation (2) was significant overall (*F*_(5,438)_ = 37.16, *p* < 0.001). Job stress (*β* = −0.19, *p* < 0.001) and job burnout (*β* = −0.38, *p* < 0.001) both predicted job satisfaction, indicating that psychological resilience moderated the first stage of the indirect effect of job stress on job satisfaction through job burnout.

**Figure 2:**
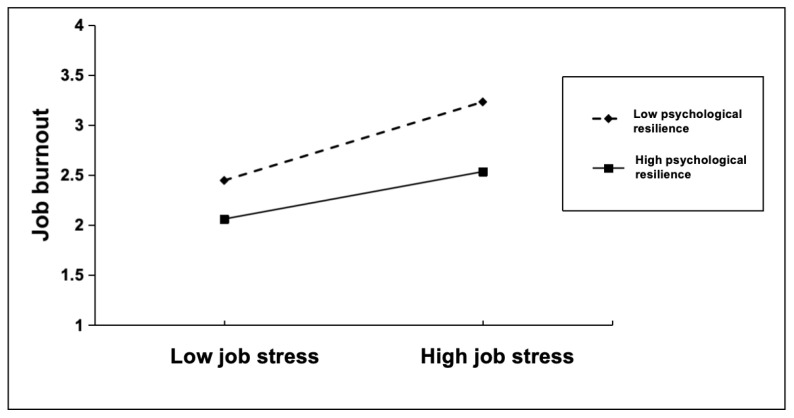
Simple slope trajectories of the moderating effect of psychological resilience on the first half of the mediation model.

To further examine the moderating effect of psychological resilience on the relationship between job stress and job burnout, this study divided the high and low levels of psychological resilience using the mean plus or minus 1 standard deviation (*M* ± 1*SD*) as the criterion, and conducted a simple slope analysis. The results showed (Figure 2) that in the low psychological resilience group, job stress positively predicted job burnout (*β*_simple_ = 0.61, *t* = 9.42, *p* < 0.001, 95% CI [0.48, 0.73]). In the high psychological resilience group, the positive predictive effect of job stress on job burnout remained evident but was considerably weaker (*β*_simple_ = 0.37, *t* = 9.07, *p* < 0.001, 95% CI [0.29, 0.45]), indicating that the positive association between job stress and job burnout was stronger among teachers with lower psychological resilience and weaker among those with higher psychological resilience.

Further analysis of conditional indirect effects under different levels of psychological resilience showed that the conditional indirect effect was −0.23 (95% CI [−0.31, −0.17]) for teachers with low psychological resilience, and −0.14 (95% CI [−0.20, −0.09]) for teachers with high psychological resilience. The difference in mediating effects between the two groups was 0.09 (*SE* = 0.03, 95% CI [0.03, 0.16]), with no zero in the confidence interval, indicating a moderated mediation effect. These results indicate that the conditional indirect effect of job stress on job satisfaction through job burnout becomes weaker as psychological resilience increases.

### 4.6. Supplementary Analysis by School Stage

To examine whether primary and secondary school physical education teachers differed in the core study variables, independent-samples *t* tests were conducted. For the supplementary analyses, junior and senior high school physical education teachers were combined into a single secondary school subgroup. The results showed that there were no significant differences between the two groups in job stress (*t* = −0.800, *p* = 0.424), job burnout (*t* = 0.028, *p* = 0.978), psychological resilience (*t* = 0.566, *p* = 0.572), or job satisfaction (*t* = 0.905, *p* = 0.366), indicating that the two groups were comparable in the average levels of the four core variables. In addition, the mediation and moderated mediation models were re-estimated separately for the two groups. The mediation model showed a similar pattern across primary and secondary school physical education teachers: in both groups, job stress positively predicted job burnout, job burnout negatively predicted job satisfaction, and the indirect effect of job stress on job satisfaction through job burnout was supported. However, the supplementary analyses suggested some variation in the moderating role of psychological resilience across school stages. Specifically, the interaction between job stress and psychological resilience, as well as the index of moderated mediation, was significant in the primary school subgroup, whereas these effects did not reach statistical significance in the secondary school subgroup. Therefore, the proposed model showed a broadly similar mediating pattern across school stages, whereas the moderating role of psychological resilience was evident only in the primary school subgroup.

## 5. Discussion

The negative association between job stress and job satisfaction suggests that sustained occupational demands may undermine physical education teachers’ positive evaluations of their work and their broader occupational well-being. This is consistent with earlier studies (Chaplain, 1995; Bhatti et al., 2011) and broadly in line with more recent review evidence indicating that teachers’ job satisfaction is closely related to broader indicators of occupational well-being and work-related conditions (Wartenberg et al., 2023). In this sense, the present study extends this line of research to primary and secondary school physical education teachers, a group whose occupational well-being may be especially vulnerable under demanding and relatively under-resourced school conditions. Although Self-Determination Theory (SDT) was not the primary framework used to develop the hypotheses, it provides a useful supplementary perspective for interpreting this pattern. When teachers’ three psychological needs of autonomy, competence, and relatedness are satisfied, it is more likely that they will demonstrate positive role behaviors and experience higher occupational well-being (Deci & Ryan, 1985). Physical education teachers often face heavy class loads, limited staffing, low professional status, and constrained career development, all of which may frustrate their basic psychological needs. Administrative arrangements may limit their autonomy in planning instruction, heavy teaching and non-teaching workloads may hinder the development of competence, and the long-standing marginalization of physical education may weaken their sense of relatedness within the school context. Accordingly, the present finding may be understood not only as evidence of a negative association between job stress and job satisfaction, but also as suggesting that demanding working conditions may be related to reduced psychological need fulfillment and lower occupational well-being among physical education teachers.

The partial mediating role of job burnout suggests that the association between job stress and job satisfaction may operate through teachers’ gradual emotional and motivational depletion. Compared with teachers of general subjects, primary and secondary school physical education teachers face more complex stressors, such as inadequate sports facilities and equipment, heavy non-teaching tasks, and multiple practical teaching responsibilities, which may make burnout particularly relevant to their work-related well-being. Previous studies have shown that job stress is positively associated with job burnout (X. Li et al., 2025) and that job burnout is negatively associated with job satisfaction (Panagopoulos et al., 2014). More importantly, a meta-analysis by Böke and Norman (2022) also reported a negative overall association between job satisfaction and burnout among physical education and sports teachers. In the present study, the correlation between job burnout and job satisfaction was −0.50, which is directionally consistent with that meta-analytic conclusion but somewhat stronger in magnitude than the overall random-effects estimate reported by Böke and Norman (2022). This difference should be interpreted cautiously rather than taken as direct evidence of overestimation, because the meta-analytic estimate reflects an average effect across multiple studies and contexts, whereas the present result is derived from a single cross-sectional sample of Chinese primary and secondary school physical education teachers. The somewhat stronger association observed here may therefore be related to the relatively homogeneous occupational context of physical education teachers, the specific institutional pressures they face, and the use of self-report measures collected at the same time point. As previously stated, the JD-R model adopted in this study suggests that job demands and job resources jointly determine the balance of individual resources, thereby influencing employees’ work attitudes and behaviors (Demerouti et al., 2001). When job demands remain high while available resources are insufficient, teachers may be more likely to experience energy depletion and emotional exhaustion (X. Li et al., 2025). In this sense, job burnout may be understood as one psychological pathway through which job stress is associated with lower job satisfaction among physical education teachers. In the work context of primary and secondary school physical education teachers, when multiple stresses interweave, insufficient personal and organizational resources may be associated with higher burnout and lower job satisfaction, whereas stronger resource reserves may help preserve occupational well-being.

The moderating pattern observed in the overall sample suggests that personal psychological resources may shape how strongly job stress is associated with job burnout among primary and secondary school physical education teachers. More specifically, higher psychological resilience was associated with a weaker yet still positive relationship between job stress and job burnout in the overall sample, suggesting that resilience may attenuate, rather than eliminate, the adverse effects of job stress in demanding work contexts. This pattern can also be understood in light of the resource investment principle in COR theory, which suggests that individuals must invest resources in order to prevent resource loss, recover from losses, or acquire new resources (Hobfoll et al., 2018). In the present context, psychological resilience may be understood as an important personal resource, and higher resilience may be related to stronger coping capacity and better adaptation in stressful situations (Deng et al., 2020). Previous studies have shown that individuals with high psychological resilience tend to be less affected by environmental stress and to demonstrate higher self-efficacy during coping (Major et al., 1998), which may help reduce resource depletion among physical education teachers when confronted with stressors such as occupied class hours, inadequate equipment, and safety responsibilities. Therefore, the JD-R model and COR theory together help explain why psychological resilience may be related to a weaker stress–burnout linkage. Because psychological resilience moderated the first-stage path from job stress to job burnout, the indirect effect of job stress on job satisfaction through job burnout was also conditional on teachers’ levels of psychological resilience. Specifically, physical education teachers with higher psychological resilience may be better able to mobilize positive psychological resources when faced with job-related resource depletion, recover more quickly, and continue engaging in subsequent tasks, thereby becoming less likely to report burnout-related experiences. Supplementary analyses further indicated that this moderating role was more evident among primary school physical education teachers, whereas it did not reach statistical significance in the secondary school subgroup. This finding suggests that the moderating role of psychological resilience may vary across school stages. At the same time, this finding should not be interpreted as suggesting that responsibility for occupational well-being rests primarily with individual teachers. Although psychological resilience may serve as a beneficial personal resource, its role should be understood as complementary to, rather than a substitute for, structural and organizational improvements in teachers’ working conditions.

### Limitations and Implications

The findings of this study deepen understanding of the mechanisms underlying job satisfaction among primary and secondary school physical education teachers and provide a useful basis for future intervention efforts. Nevertheless, several limitations should be acknowledged. First, although the present model helps clarify the roles of job burnout and psychological resilience, it captures only part of the process through which job stress is associated with job satisfaction. In particular, the proposed model explained a moderate proportion of the variance in job satisfaction (*R*^2^ = 0.30), indicating that substantial variance remains attributable to factors not included in the present study. Future research may therefore incorporate additional personal, organizational, and contextual variables, such as professional identity and social support, to provide a more comprehensive explanation of job satisfaction among primary and secondary school physical education teachers. Second, because this study relied on cross-sectional data, causal relationships cannot be established. In addition, although the common method bias test did not indicate a serious problem, the use of self-report measures means that social desirability bias cannot be completely ruled out. Participants may have been inclined to present themselves in a more favorable way when reporting work-related attitudes and psychological characteristics. These design features may also have contributed to somewhat stronger observed associations among the self-reported variables. Future studies may address these issues by adopting longitudinal designs, incorporating multi-source assessments, or including a dedicated measure of social desirability. Third, because no additional exclusion criterion was imposed based on teaching experience or substitute teacher status, the sample may have included teachers at very early career stages or under different employment arrangements, which may have introduced additional heterogeneity into the findings. Future research may adopt more fine-grained sampling criteria to examine whether the observed relationships differ across career stages or employment types. Finally, although supplementary analyses were conducted separately for primary and secondary school teachers, these subgroup analyses were exploratory in nature and did not involve formal cross-group parameter equality tests. Future research may adopt more rigorous multi-group approaches to further examine whether the proposed model differs across school stages.

Despite these limitations, several practical implications can still be drawn from the present findings. First, schools should not only allocate class hours more appropriately, but also establish a workload accounting system that formally recognizes the non-teaching duties uniquely undertaken by physical education teachers, such as organizing break-time exercises, supervising after-school physical activity, coaching school sports teams, preparing students for competitions, and managing event-related safety and logistics. These duties should be incorporated into formal workload calculations and linked to time compensation, reduced teaching loads, or performance recognition. Second, schools should provide targeted organizational support for these recurring duties. For example, before major school sports events or interschool competitions, schools may reserve protected preparation time, reduce overlapping administrative tasks, and provide assistance with scheduling, equipment preparation, student management, and safety documentation. Third, material resources and emotional support within the school may serve as important forms of tangible and intangible support, thereby strengthening physical education teachers’ sense of belonging and organizational identification. Enhancing the professional status of physical education teachers and recognizing the importance of their work may further support the fulfillment of their basic psychological needs. When these needs are met, physical education teachers are more likely to demonstrate positive work attitudes and behaviors, along with higher job satisfaction and professional competence (Deci & Ryan, 2000). Additionally, schools can help teachers build positive cognitive patterns and enhance psychological resource reserves by conducting stress coping workshops and establishing psychological resilience training courses (such as mindfulness training and problem-solving skills), enabling them to cope with workplace challenges with optimistic, positive, and resilient mental qualities. The supplementary findings also suggest that resilience-oriented support strategies may be particularly relevant for primary school physical education teachers, although stage-specific intervention effects should be examined more directly in future research. Accordingly, resilience-oriented interventions should not be treated as a substitute for improving school-level conditions. Rather, they should be implemented alongside efforts to reduce excessive job demands, improve resource allocation, and strengthen institutional support for physical education teachers.

## 6. Conclusions

This study shows that job stress among primary and secondary school physical education teachers exerts a considerable influence on their job satisfaction. The findings underscore that, beyond the direct effects of stress, educational institutions must attend to the detrimental role of job burnout in diminishing satisfaction. Accordingly, targeted interventions should be implemented to cultivate teachers’ psychological resilience, but such efforts should be accompanied by school-level changes aimed at reducing excessive job demands and improving working conditions. Such measures may help enhance job satisfaction, support physical education teachers’ professional development, and, in turn, promote adolescents’ healthy development.

By constructing a moderated mediation model, this study advances theoretical understanding of physical education teachers’ occupational mental health. It offers practical implications for educational administrators seeking to optimize working environments, design evidence-based stress interventions, and foster psychological resilience. Reducing job burnout and improving job satisfaction may promote teachers’ retention and well-being while also supporting high-quality teaching and student development. Ultimately, these efforts contribute to the advancement of school physical education, the holistic development of adolescents, and the overall quality of education.

## Figures and Tables

**Table 1:** Means, standard deviations, and correlation coefficients of variables.

	1	2	3	4	5	6	7	8
1. Age group	1.00							
2. Marital status	−0.71 **	1.00						
3. Teaching experience	0.91 **	−0.67 **	1.00					
4. Education level	−0.43 **	0.38 **	−0.46 **	1.00				
5. Job stress	−0.01	0.07	−0.02	0.01	1.00			
6. Job satisfaction	0.19 **	−0.17 **	0.16 **	−0.05	−0.40 **	1.00		
7. Job burnout	−0.11 *	0.10 *	−0.11 *	0.11 *	0.53 **	−0.50 **	1.00	
8. Psychological resilience	0.15 **	−0.13 **	0.13 **	0.02	−0.26 **	0.75 **	−0.52 **	1.00
*M*	2.91	1.35	2.43	2.03	3.05	3.54	2.59	3.87
*SD*	1.29	0.48	1.45	0.55	0.88	0.85	0.65	0.75

Notes. Age group: 1 = 18–25 years old, 2 = 26–30 years old, 3 = 31–40 years old, 4 = 41–50 years old, 5 = 51–60 years old; Marital status: 1 = married, 2 = unmarried; Teaching experience: 1 = less than 5 years, 2 = 6–10 years, 3 = 11–20 years, 4 = 21–30 years, 5 = more than 31 years; Education level: 1 = college graduates, 2 = undergraduate, 3 = postgraduate and above; * *p* < 0.05, ** *p* < 0.01.

**Table 2:** The mediating effect of job burnout between job stress and job satisfaction.

Mediation Effects	Completely Standardized Effect	Bootstrap SE	95% CI		Proportion of Total Effect
			LLCI	ULCI	
Total effect of job stress on job satisfaction	−0.397	0.043	−0.481	−0.312	
Direct effect of job stress on job satisfaction	−0.194	0.047	−0.288	−0.101	48.87%
Mediating effect of job burnout in the path from job stress to job satisfaction	−0.203	0.031	−0.264	−0.144	51.13%

Notes. Because job stress, job burnout, and job satisfaction were standardized prior to analysis, the reported total, direct, and indirect effects are completely standardized effect sizes.

**Table 3:** The moderating effect of psychological resilience on the path from job stress to job burnout.

Regression Equation		Overall Fit Indices			Significance of Regression Coefficients				
Outcome Variable	Predictor Variable	*R*	*R* ^2^	*F*	*β*	*SE*	*t*	95% CI	
								LLCI	ULCI
Equation (1) job burnout		0.68	0.46	61.97 ***					
	Age group				0.02	0.07	0.22	−0.12	0.15
	Marital status				−0.08	0.11	−0.75	−0.29	0.13
	Teaching experience				−0.05	0.06	−0.92	−0.16	0.06
	Job stress				0.49	0.04	11.97 ***	0.41	0.57
	Psychological resilience				−0.42	0.04	−11.34 ***	−0.49	−0.35
	Job stress × Psychological resilience				−0.12	0.04	−3.41 ***	−0.19	−0.05
Equation (2) job satisfaction		0.55	0.30	37.16 ***					
	Age group				0.15	0.08	1.89	−0.01	0.30
	Marital status				−0.07	0.12	−0.62	−0.31	0.16
	Teaching experience				−0.05	0.06	−0.83	−0.18	0.07
	Job stress				−0.19	0.05	−4.10 ***	−0.29	−0.10
	Job burnout				−0.38	0.05	−8.03 ***	−0.48	−0.29

Notes. LLCI = lower limit of the 95% confidence interval; ULCI = upper limit of the 95% confidence interval. *** *p* < 0.001.

## Data Availability

The data presented in this study are available on request from the corresponding author due to ethical restrictions and participant confidentiality.

## References

[B1-behavsci-16-00737] Agyapong B., Obuobi-Donkor G., Burback L., Wei Y. (2022). Stress, burnout, anxiety and depression among teachers: A scoping review. International Journal of Environmental Research and Public Health.

[B2-behavsci-16-00737] Bakker A. B., Demerouti E., Taris T. W., Schaufeli W. B., Schreurs P. J. (2003). A multigroup analysis of the job demands-resources model in four home care organizations. International Journal of Stress Management.

[B3-behavsci-16-00737] Bhatti N., Hashmi M. A., Raza S. A., Shaikh F. M., Shafiq K. (2011). Empirical analysis of job stress on job satisfaction among university teachers in Pakistan. International Business Research.

[B4-behavsci-16-00737] Böke H., Norman G. (2022). The relationship between job satisfaction and professional burnout: A systematic review and meta-analysis on physical education and sports teachers. International Journal of Eurasian Education and Culture.

[B5-behavsci-16-00737] Chaplain R. P. (1995). Stress and job satisfaction: A study of English primary school teachers. Educational Psychology.

[B6-behavsci-16-00737] Deci E. L., Ryan R. M. (1985). The general causality orientations scale: Self-determination in personality. Journal of Research in Personality.

[B7-behavsci-16-00737] Deci E. L., Ryan R. M. (2000). The “what” and “why” of goal pursuits: Human needs and the self-determination of behavior. Psychological Inquiry.

[B8-behavsci-16-00737] Demerouti E., Bakker A. B., Nachreiner F., Schaufeli W. B. (2001). The job demands-resources model of burnout. Journal of Applied Psychology.

[B9-behavsci-16-00737] Deng Q., Zheng B., Chen J. (2020). The relationship between personality traits, resilience, school support, and creative teaching in higher school physical education teachers. Frontiers in Psychology.

[B10-behavsci-16-00737] Den Hartigh R., Hill Y. (2022). Conceptualizing and measuring psychological resilience: What can we learn from physics?. New Ideas in Psychology.

[B11-behavsci-16-00737] Eirín-Nemiña R., Sanmiguel-Rodríguez A., Rodríguez-Rodríguez J. (2022). Professional satisfaction of physical education teachers. Sport, Education and Society.

[B12-behavsci-16-00737] Ferry M., Westerlund R. (2023). Professional networks, collegial support, and school leaders: How physical education teachers manage reality shock, marginalization, and isolation in a decentralized school system. European Physical Education Review.

[B13-behavsci-16-00737] Ghasemi F., Beversdorf D. Q., Herman K. C. (2024). Stress and stress responses: A narrative literature review from physiological mechanisms to intervention approaches. Journal of Pacific Rim Psychology.

[B14-behavsci-16-00737] Hayes A. F. (2012). PROCESS: A versatile computational tool for observed variable mediation, moderation, and conditional process modeling *[White paper]*.

[B15-behavsci-16-00737] Hobfoll S. E. (2002). Social and psychological resources and adaptation. Review of General Psychology.

[B16-behavsci-16-00737] Hobfoll S. E., Halbesleben J., Neveu J. P., Westman M. (2018). Conservation of resources in the organizational context: The reality of resources and their consequences. Annual Review of Organizational Psychology and Organizational Behavior.

[B17-behavsci-16-00737] Hoppock R. (1935). Job satisfaction.

[B18-behavsci-16-00737] Kliziene I., Cizauskas G., Sipaviciene S., Aleksandraviciene R., Zaicenkoviene K. (2021). Effects of a physical education program on physical activity and emotional well-being among primary school children. International Journal of Environmental Research and Public Health.

[B19-behavsci-16-00737] Kougioumtzis K., Patriksson G., Stråhlman O. (2011). Physical education teachers’ professionalization: A review of occupational power and professional control. European Physical Education Review.

[B20-behavsci-16-00737] Kyriacou C., Sutcliffe J. (1977). Teacher stress: A review. Educational Review.

[B21-behavsci-16-00737] Li Q., Pei L., Wu D. (2014). Teacher resilience: Construct and influential factors. Journal of Educational Studies.

[B22-behavsci-16-00737] Li S. (2023). The effect of teacher self-efficacy, teacher resilience, and emotion regulation on teacher burnout: A mediation model. Frontiers in Psychology.

[B23-behavsci-16-00737] Li S., Wang Y. (2024). The effect of job stress on secondary school physical education teachers’ work engagement: The mediating role of self-efficacy. Psychology in the Schools.

[B24-behavsci-16-00737] Li X., Xu H., Zhang J., Cao X. (2025). The relationship between occupational stress and burnout among primary and secondary school physical education teachers: Evidence from multiverse-style analysis and diary method. BMC Psychology.

[B25-behavsci-16-00737] Lin X. Q., Yin H. C., Yang J. R., Chen Y. R. (2005). Research on source structural model of occupational pressure of primary and middle school gym teachers. China Sport Science.

[B26-behavsci-16-00737] Major B., Richards C., Cooper M. L., Cozzarelli C., Zubek J. (1998). Personal resilience, cognitive appraisals, and coping: An integrative model of adjustment to abortion. Journal of Personality and Social Psychology.

[B28-behavsci-16-00737] Ma’mun A., Tinaz C., Anira A., Syarifatunnisa S., Hertem Ö. O., Mahendra A., Juliantine T. (2025). Physical education and school sport in emerging nations: A comparison of Indonesia and Türkiye. Frontiers in Sports and Active Living.

[B27-behavsci-16-00737] Maslach C., Schaufeli W. B., Leiter M. P. (2001). Job burnout. Annual Review of Psychology.

[B29-behavsci-16-00737] Michie S. (2002). Causes and management of stress at work. Occupational and Environmental Medicine.

[B30-behavsci-16-00737] Nelson D. R., Adger W. N., Brown K. (2007). Adaptation to environmental change: Contributions of a resilience framework. Annual Review of Environment and Resources.

[B31-behavsci-16-00737] Panagopoulos N., Anastasiou S., Goloni V. (2014). Professional burnout and job satisfaction among physical education teachers in Greece. Journal of Scientific Research & Reports.

[B32-behavsci-16-00737] Podsakoff P. M., MacKenzie S. B., Lee J. Y., Podsakoff N. P. (2003). Common method biases in behavioral research: A critical review of the literature and recommended remedies. Journal of Applied Psychology.

[B33-behavsci-16-00737] Richards K. A. R., Gaudreault K. L., Woods A. M. (2017a). Understanding physical educators’ perceptions of mattering: Validation of the perceived mattering questionnaire-physical education. European Physical Education Review.

[B34-behavsci-16-00737] Richards K. A. R., Levesque-Bristol C., Templin T. J., Graber K. C. (2016). The impact of resilience on role stressors and burnout in elementary and secondary teachers. Social Psychology of Education.

[B35-behavsci-16-00737] Richards K. A. R., Washburn N., Carson R. L., Hemphill M. A. (2017b). A 30-year scoping review of the physical education teacher satisfaction literature. Quest.

[B36-behavsci-16-00737] Riyadi S. (2015). Effect of work motivation, work stress and job satisfaction on teacher performance at senior high school (SMA) throughout The State Central Tapanuli, Sumatera. IOSR Journal of Humanities and Social Science.

[B37-behavsci-16-00737] Sáenz-López P., Almagro B. J., Ibáñez S. J. (2011). Describing problems experienced by Spanish novice physical education teachers. Open Sports Sciences Journal.

[B38-behavsci-16-00737] Selye H. (1976). Stress without distress. Psychopathology of human adaptation.

[B39-behavsci-16-00737] Skaalvik E. M., Skaalvik S. (2017). Dimensions of teacher burnout: Relations with potential stressors at school. Social Psychology of Education.

[B40-behavsci-16-00737] Travers C. (2017). Current knowledge on the nature, prevalence, sources and potential impact of teacher stress. Educator stress: An occupational health perspective.

[B41-behavsci-16-00737] Trigueros R., Aguilar-Parra J. M., Cangas A. J., López-Liria R., Álvarez J. F. (2019). Influence of physical education teachers on motivation, embarrassment and the intention of being physically active during adolescence. International Journal of Environmental Research and Public Health.

[B42-behavsci-16-00737] Wang J., Liu A., Cai Y., Sun Y. (2025). Resilience: A mediator between teachers’ personal resources, contextual resources and positive outcomes of well-being and commitment. International Journal of Educational Research.

[B43-behavsci-16-00737] Wanous J. P., Lawler E. E. (1972). Measurement and meaning of job satisfaction. Journal of Applied Psychology.

[B44-behavsci-16-00737] Wartenberg G., Aldrup K., Grund S., Klusmann U. (2023). Satisfied and high performing? A meta-analysis and systematic review of the correlates of teachers’ job satisfaction. Educational Psychology Review.

[B45-behavsci-16-00737] Wen Z., Ye B. (2014). Different methods for testing moderated mediation models: Competitors or backups?. Acta Psychologica Sinica.

[B46-behavsci-16-00737] Xanthopoulou D., Bakker A. B., Demerouti E., Schaufeli W. B. (2007). The role of personal resources in the job demands-resources model. International Journal of Stress Management.

[B47-behavsci-16-00737] Xu Z. Y., Zhao Z. H. (2012). An empirical study of job satisfaction of Beijing primary school teachers. Teacher Education Research.

[B48-behavsci-16-00737] Yan J., Dai X. (2025). Double marginalization: An ethnographic-ecological analysis of rural PE teachers’ professional development between urban and underdeveloped areas. Frontiers in Public Health.

[B49-behavsci-16-00737] Yan J., Dai X., Xuecui B. I. (2025). How faculty support impact physical education teachers’ career satisfaction: A chain mediation model. Acta Psychologica.

[B50-behavsci-16-00737] Yang X., Ma B. J., Chang C. L., Shieh C. J. (2014). Effects of workload on burnout and turnover intention of medical staff: A study. Studies on Ethno-Medicine.

[B51-behavsci-16-00737] Zhou H., Xu S. Q., Chung D. H., Dang D. X. (2025). Job satisfaction mediates the effect of self-efficacy on work engagement among physical education teachers in economically disadvantaged areas. PLoS ONE.

